# AI-guided prefusion stabilization of the human coronavirus OC43 spike protein enables universal embecovirus antigen design

**DOI:** 10.1371/journal.ppat.1013998

**Published:** 2026-03-23

**Authors:** Jelle M. Melchers, Jarek Juraszek, Ruben J.G. Hulswit, Daan van Overveld, Lam Le, Frank J.M. van Kuppeveld, Daniel L. Hurdiss, Berend-Jan Bosch, Johannes P.M. Langedijk, Mark J.G. Bakkers

**Affiliations:** 1 Virology Section, Infectious Diseases and Immunology Division, Department of Biomolecular Health Sciences, Faculty of Veterinary Medicine, Utrecht University, Utrecht, The Netherlands; 2 Janssen Vaccines & Prevention BV, Leiden, The Netherlands; 3 ForgeBio BV, Amsterdam, The Netherlands; Institut Pasteur, FRANCE

## Abstract

The continued threat of zoonotic coronavirus spillovers underscores the need for cross-species applicable vaccine design strategies. The genus Embecovirus includes human coronaviruses OC43 and HKU1 as well as relevant veterinary pathogens. The coronavirus spike (S) fusion glycoprotein, key to viral entry and protective immunity, is inherently metastable, complicating vaccine development. Using the ReCaP AI tool, we stabilized the prefusion conformation of OC43 S through rationally combined amino acid substitutions, resulting in markedly enhanced expression and thermal stability. The substitutions were transferable to equine coronavirus (ECoV) S and HKU1. Cryo-EM structures of stabilized OC43 and ECoV S revealed that stabilization was achieved by arresting the release of the fusion peptide and keeping the S1^B^ receptor binding domain in the ‘down’ state by improving the complex polar interactions of neighboring S1^B^ domains and the bound free fatty acid at the interprotomer S1^B^ interface. This work provides the first ECoV S structure and a broadly applicable framework for engineering stabilized Embecovirus S antigens.

## Author summary

Coronaviruses pose a constant threat to global health due to the potential transmission from animal to human hosts. Vaccine development has been limited by the instability of the coronavirus spike protein in the prefusion state and since future spillover events may involve previously unidentified coronaviruses there is a need for broadly applicable stabilization strategies. In this study, we describe an approach to stabilize embecovirus prefusion spike proteins using the human coronavirus OC43 spike as a prototype. By combining phylogenetic analysis of OC43 laboratory strains, structure-guided predictions using the machine-learning algorithm ReCaP, and rational structure-based design, we engineered spike proteins with improved prefusion stability. Importantly, these stabilizing features were transferable to other embecoviruses, including equine coronavirus (ECoV) and human coronavirus HKU1. We further determined high-resolution cryo–electron microscopy structures of the stabilized OC43 and ECoV spikes, confirming preservation of the prefusion conformation and providing insights into the mechanism by which the substitutions improve stability. Together, our results establish a generalizable framework for stabilizing embecovirus spike proteins and support pandemic preparedness efforts to mitigate future zoonotic spillover events.

## Introduction

**Fig 1:**
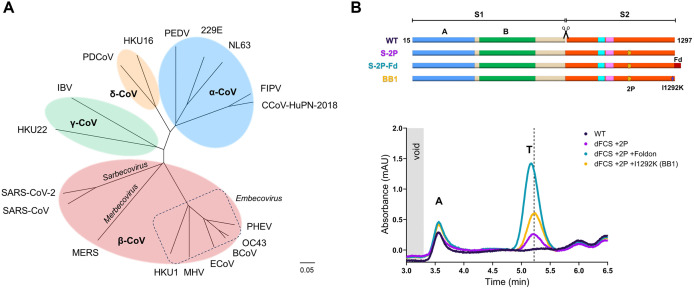
Design of an OC43 S backbone for screening purposes. **(A**) Maximum-likelihood phylogenetic tree based on amino acid sequences of coronavirus S proteins. **(B)** (Top) Schematic of OC43 S ectodomain variants with indicated stabilizing elements; furin cleavage site at the S1/S2 border (scissors), A1075P and L1076P substitutions (2P), T4 fibritin foldon domain (Fd), and I1292K substitution. Subdomains S1^A^ (blue), S1^B^ (green), fusion peptide-proximal region (cyan) and fusion peptide (magenta) are shown. (Bottom) Analytical SEC profiles measured from supernatants of Expi293 cells transfected with OC43 S variants from the top panel. The dashed line at 5.2 minutes indicates the expected retention time of OC43 S trimers without a foldon trimerization domain. ‘T’ and ‘A’ indicate trimer and aggregate peaks respectively.

Coronaviruses are a large family of enveloped, positive-sense single-stranded RNA viruses that infect a wide range of mammalian and avian hosts, including humans. The recurrent introduction of coronaviruses into human populations, exemplified by the emergence of severe acute respiratory syndrome coronavirus (SARS-CoV), Middle East respiratory syndrome coronavirus (MERS-CoV), and SARS-CoV-2 over the past 25 years, underscores the importance of vigilant surveillance and pandemic preparedness [1–3]. *Embecovirus,* a subgenus within the genus *Betacoronavirus*, includes viruses of significant relevance to both veterinary and human health (Fig 1A). Human coronaviruses OC43 and HKU1, both members of this subgenus, are commonly associated with mild to moderate respiratory infections. However, despite their typically benign nature, both viruses can pose significant health risks to vulnerable populations, particularly the elderly and immunocompromised individuals [4,5]. Embecoviruses are also of veterinary importance, with equine coronavirus (ECoV), bovine coronavirus (BCoV) and porcine hemagglutinating encephalomyelitis virus (PHEV) causing diseases with considerable economic burden in their respective hosts [6–8]. Currently, no vaccines or antivirals are available to prevent or treat embecovirus infections.

A central determinant of coronavirus pathogenicity and infectivity is the spike (S) protein, which mediates virus entry into host cells [9]. The S protein is a class I fusion protein that is composed of two subunits: S1, responsible for receptor binding and S2, which mediates membrane fusion [10,11]. Within the S1 subunit, domains A and B (S1^A^ and S1^B^) (Fig 1B, upper panel) generally bind to respectively glycan and proteinaceous receptors [12–15]. Transition of the S1^B^ domain from a ‘down’ (closed) state to an ‘up’ (receptor accessible) state is thought to occur either spontaneously, in case of viruses like MERS-CoV, SARS-CoV, SARS-CoV-2, and PEDV [16–18], or may require specific cues, as was recently described for HKU1 [19]. Dynamics of S1^B^ transitioning between ‘up’ and ‘down’ conformations seem to be further regulated in spike proteins of betacoronaviruses by binding of fatty acids in a conserved hydrophobic pocket in S1^B^, which locks the spike in a 3-down conformation [20–23].

During biogenesis or upon binding to the host cell receptor, host proteases prime the homotrimeric S protein for fusion by processing at S1/S2 and S2’ cleavage sites [24]. This proteolytic processing allows the release of the S1 domains during entry, which serve as a fusion-suppressive cap over the S2 subunit [25,26]. After S1 release, the S2 domain undergoes a dramatic conformational change from a metastable prefusion, to a highly stable postfusion conformation, enabling merging of viral and cellular membranes and release of the viral genome into the host cell cytoplasm [27].

The prefusion conformation of the S protein is critically important for vaccine development and antiviral strategies, as it represents the functional form of the protein and is the target for neutralizing antibodies [28–30]. However, the prefusion conformation is difficult to express, hindering structural studies and limiting its use as a vaccine immunogen. Previously, prefusion S stabilization has been described for SARS-CoV, MERS-CoV and SARS-CoV-2 by mutation of the S1/S2 furin cleavage site, introduction of a double proline substitution in the S2 hinge region, as well as various species-specific approaches [30–36]. Whereas most of these approaches were based on soluble ectodomains fused to a heterologous T4 fibritin foldon trimerization domain, a closed prefusion S protein was described for SARS-CoV-2 that was no longer dependent on foldon [33]. While foldon presents an effective method of trimerization, vaccines equipped with this domain have been shown to induce foldon-specific antibodies that increase upon repeated vaccination [37]. The long-term effects on vaccine efficacy and potential risks associated with the induction of non-relevant anti-foldon antibodies remain unknown. Additionally, studies indicate that immunization with a prefusion stabilized, S1^B^ ‘closed’ SARS-CoV-2 spike induces a more potent neutralizing immune response when compared to spike antigens which adopt a more ‘open’ trimer [38,39]. Notably, a fully ‘locked’ spike unable to adopt the up conformation induced comparable neutralizing titers, although with reduced breadth indicating that transient exposure of S1^B^ is important to elicit antibodies against more conserved epitopes.

Here, we describe stabilization of the prefusion conformation of the OC43 S protein ectodomain using a combination of rational, phylogenetic and AI-guided techniques. We demonstrate that our stabilization strategy is transferable across embecoviruses, applying it successfully to the spikes of both HKU1 and ECoV. To understand the mechanism of stabilization, we solved the structures of the stabilized OC43 and ECoV S proteins using cryogenic electron microscopy (cryo-EM), marking the first structural characterization of ECoV S. The results provide valuable insights into the spike structure-function relationship and will pave the way for the development of more efficacious vaccines. Together, our findings highlight the potential of this approach for broad-spectrum vaccine design against embecoviruses.

## Results

### Generation of a backbone for screening purposes

To screen for substitutions that can stabilize the prefusion conformation of OC43 S we first designed several potential backbones that would allow for sensitive detection of both expression-increasing and expression-decreasing substitutions. These constructs encode the S ectodomain (residues 1–1297) based on a 2014 clinical isolate and differed in the removal of the S1/S2 furin cleavage site (dFCS), introduction of a double proline substitution (2P; A1075P and L1076P) in Heptad Repeat 1 (HR1), and the presence of a heterologous foldon trimerization domain (Fig 1B, upper panel). Backbone constructs were transiently expressed in Expi293F cells and cell supernatants were analyzed by analytical size-exclusion chromatography (SEC) (Fig 1B, lower panel). A wild-type construct lacking stabilizing modifications (WT) gave no detectable expression of S trimers. A spike combining furin site deletion with the 2P substitutions (S-2P) led to a peak at the expected retention time of a prefusion S trimer. Further addition of a C-terminal foldon (S-2P-Fd) resulted in a slightly shorter retention time, and boosted S expression levels sixfold. While heterologous foldon or GCN4 domains are commonly used to drive trimerization of the spike monomer [17,25,26,30,40], their immunogenicity raises concerns over off-target immune responses [37]. To circumvent this, we designed an I1292K substitution in the C-terminal part of the HR-2 stem helix to improve local trimeric coiled-coil interactions. This minimal backbone construct (BB1) had a 2-fold higher trimer expression compared to parental S-2P, but still a lower expression than the foldon-containing S-2P-Fd construct. The intermediate expression of BB1 was considered suitable for identifying stabilizing substitutions in subsequent screens.

### Rational stabilization of the HR2 stem region

**Fig 2:**
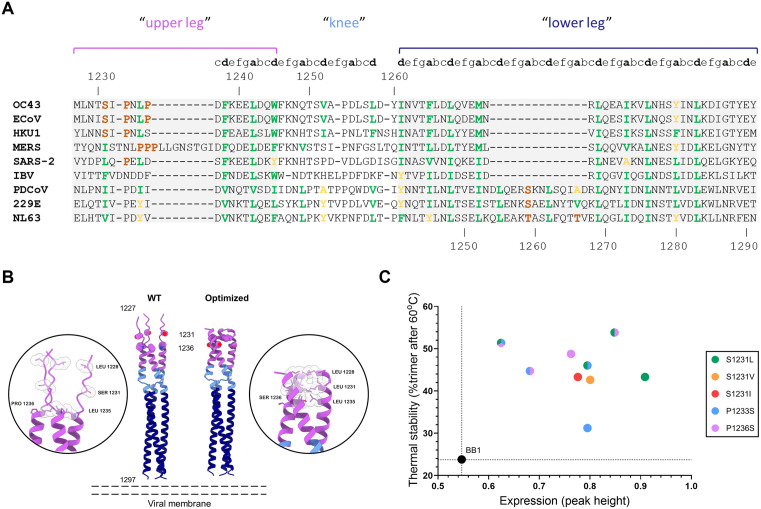
Rational design of OC43 S trimers by optimization of the stem heptad repeat. **(A)** Sequence alignment of the stem regions of coronavirus spike proteins representing alpha-CoV; HCoV-229E and HCoV-NL63, gamma-CoV; IBV, beta-CoV; HCoV-OC43, HCoV-HKU1, ECoV, MERS-CoV and SARS-CoV-2, and delta-CoV; Hu-PDCoV. Residue numbering is based on the OC43 (top) and NL63 S (bottom) sequence, respectively. “Upper leg”, “knee”, and “lower leg” parts of the stem are indicated [39]. Hydrophobic residues in heptad a and d positions are colored green, helix-breaking proline or hydrophilic serine residues are colored orange. **(B)** AlphaFold3 predicted structures of the OC43 S stem (1227-1297) in cartoon representation. Predicted models of the wild type sequence (WT), or incorporating S1231L and P1236S substitutions (‘Optimized’) are shown. Atoms of residue side chains at positions 1228, 1231 and 1235 are shown as spheres. **(C)** Expression and thermal stability of OC43 S BB1 and stem substitutions based on peak height of spike protein peaks measured by analytical SEC of clarified cell culture supernatant.

Next, we focused design efforts on the trimeric Heptad Repeat 2 (HR2) stem region at the membrane-proximal base of the S protein. The coronavirus HR2 region consists of two alpha-helical coiled-coil regions (‘upper leg’ and ‘lower leg’) connected by an unstructured ‘knee’ region (Fig 2A) [41]. As with other class I viral fusion proteins, the coiled-coil packing within HR2 must be suboptimal to permit the conformational rearrangements required for refolding into the post-fusion state [42–44]. Consistent with this, inspection of the *a* and *d* positions that line the interior of the coiled-coil revealed suboptimal residues in OC43 S: a serine at position 1231, and helix-disrupting prolines at positions 1233 and 1236. Structural modeling of the wildtype HR2 sequence using Alphafold3 [45] confirmed a break in the helical structure in the 1231–1236 region (Fig 2B). Notably, sequence comparisons with other embecoviruses revealed that these destabilizing residues are conserved across the genus (Fig 2A).

We evaluated substitution of S1231 for the hydrophobic amino acids Leu, Val or Ile, residues typically found in the hydrophobic cores of coiled-coils, as well as Ser substitutions for the helix-breaking proline residues at positions 1233 and 1236. Substitutions were made individually or in combination in the BB1 background to assess their effect on S expression and thermal stability (Fig 2C). All tested substitutions increased expression of OC43 S trimers. Substitution of S1231 for Leu gave similar stability, but a higher expression, than substituting for either Val or Ile. The individual substitution of prolines 1233 and 1236 to Ser likewise improved expression and thermal stability. The combined substitution of P1233 and P1236 resulted in a modest increase in expression of the protein. Of all the tested variants for this approach we selected the combination of S1231L with P1236S since this gave a 2-fold increase in thermal stability compared to BB1 and was among the highest in expression (Fig 2C).

Since the heptad repeat motif is commonly shared in the stem region of coronavirus spikes beyond the betacoronavirus genus, and the mechanism of helix separation during refolding to the postfusion spike is conserved [27], we aimed to also test the HR2 optimization approach to spikes belonging to the alpha- and delta-coronavirus genera. When analyzing the coiled-coil region in the spike protein of these viruses, which contain an insertion of two heptad repeats (14 amino acids) [10,46], similarly suboptimal residues are evident in heptad *a* and *d* positions (Fig 2A). Likewise, we observed improvements on expression and stability of S upon Val substitution of these residues in NL63 (T1259 and T1266), 229E (S1076), and human PDCoV (S1060 and A1067) spike proteins (S1 Fig) demonstrating that this approach is broadly transferable to the S proteins of different CoV genera.

### Screening of prefusion S stabilizing substitutions

**Fig 3:**
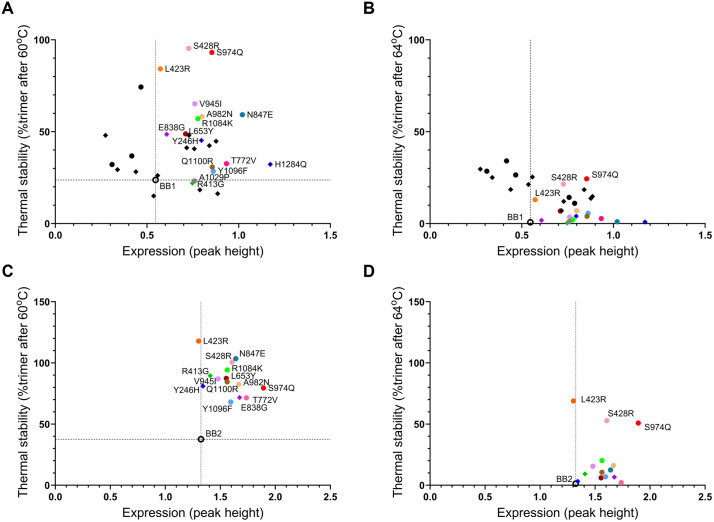
Screening of prefusion OC43 S stabilizing substitutions. **(A**) Expression and thermal stability (after 60^o^C incubation) of selected substitutions in OC43 S “BB1” as determined by analytical SEC of cell supernatant. Substitutions derived from tissue-culture adaptation are shown as diamonds, ReCAP-predicted substitutions are shown as spheres. **(B)** Expression and thermal stability of substitutions tested in (A) after supernatant was incubated at 64^o^C. **(C-D)** Expression and thermal stability of positive candidates in the BB2 background after 60^o^C **(C)**, and 64^o^C (D) incubation.

To identify additional substitutions that enhance expression and stability of prefusion OC43 S, we employed two complementary methodologies. In the first approach, we analyzed spike sequences from tissue culture-adapted OC43 strains. The ATCC VR-759 strain that is widely used to study OC43, was isolated in 1967 following a series of extensive passaging in suckling mouse brain and cell culture [47]. We hypothesized that the extensive cell culture passaging by numerous laboratories since then, often under stressful conditions such as incubation at 37°C and exposure to freeze-thaw cycles, could have selected for variants with mutations in the S protein that confer increased prefusion stability. Spike sequences of the OC43 ATCC VR-759 strain available in GenBank (S1 Table) were analyzed, and 25 mutations were identified that were prevalent in tissue culture-adapted strains but not in clinical isolates. In the second approach, we applied the previously developed AI/ML amino acid classification algorithm, ReCaP [48] to predict stabilizing substitutions based on the spike’s local residue microenvironment (PDB ID: 6NZK [15]). ReCaP mutations were chosen where a different amino acid from the input sequence was predicted, and the suggested residue differed notably in biochemical properties. We selected 14 ReCaP-predicted and 13 culture-derived substitutions for experimental validation, all of which fulfilled the additional criteria of minimal solvent exposure. Each selected substitution was individually introduced into the BB1 construct and assessed for impact on expression level and thermal stability (Fig 3A, 3B). Four culture-derived substitutions (Y246H, R413G, E838G, and H1284Q), and eleven ReCaP substitutions (L423R, S428R, L653Y, T772V, N847E, V945I, S974Q, A982N, R1084K, Y1096F, and Q1100R) were selected for follow-up, since these showed >50% expression increase or >2-fold stability improvement. Notably, H1284Q exhibited the greatest increase in trimer expression, while L423R, S428R, and S974Q demonstrated the most notable improvements in thermal stability, with nearly the entire trimer fraction persisting after incubation at 60°C. This stability improvement was further emphasized at 64°C, where L423R, S428R, and S974Q variants retained detectable trimer peaks, while no trimer peak remained for the other substitutions (Fig 3B and S2 Fig).

For additional validation, we re-evaluated the selected panel of 15 stabilizing substitutions in a second-generation backbone with higher baseline expression. We combined the highest expression increasing substitution H1284Q, with the HR2 stem substitutions S1231L, P1236S, and A1029P, a substitution analogous to A942P used in prefusion-stabilized SARS-CoV-2 spikes such as HexaPro [32] and S-closed [33]. This new backbone, termed BB2, showed a two-fold increase in expression relative to BB1 (Fig 3C). Importantly, all selected substitutions retained their positive effects on expression and/or thermal stability in the BB2 background (Fig 3C, 3D). Additional testing was done to evaluate if substitutions which were in close proximity in the structure would have a positive or negative impact when introduced together. Based on these data we concluded that Y1096F and Q1100R, separated by a single alpha helical turn, were mutually exclusive since the double substitution exhibited lower S trimer expression than the individual substitutions (S3 Fig).

### Design and characterization of lead candidates

**Fig 4:**
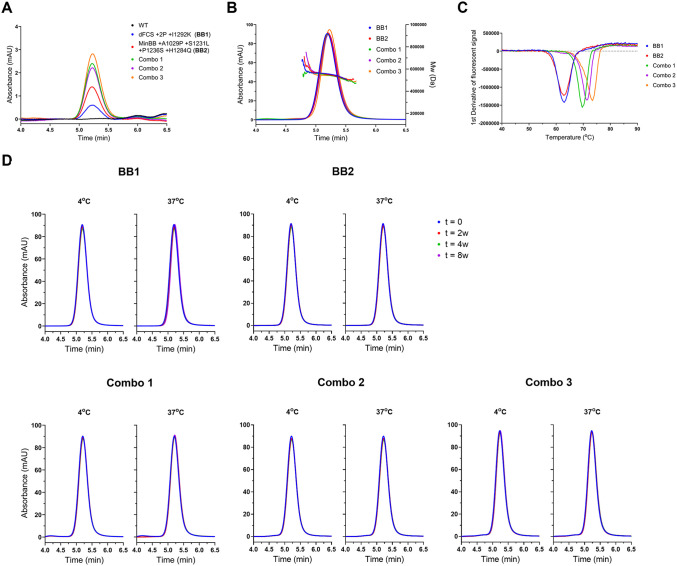
Characterization of stabilized OC43 spike proteins. **(A**) Analytical SEC profiles of clarified supernatant of Expi293F cells transfected with indicated OC43 S variants **(B)** Analytical SEC and MALS analysis of purified minimally stabilized OC43 backbone (BB1), BB2 and incremental combinations of substitutions (Combo 1, 2 and 3) **(C)** Melting temperature (Tm_50_) determination by DSF of the indicated OC43 S variants. The average of three technical replicates is shown. **(D)** Analytical SEC analysis of the long-term stability of purified BB1, BB2, Combo 1, Combo 2 and Combo 3 when stored at 4^o^C (left) and 37^o^C (right) for indicated time periods.

Next, we tested the effects of combining these mutations on expression and stability by generating three variants containing an increasing number of stabilizing substitutions (Fig 4A). Combo 1, which includes L423R, S428R, and S974Q, demonstrated a notable 1.7-fold increase in expression levels compared to the BB2 reference. In contrast, Combo 2, which added five additional mutations (N847E, E838G, A982N, R1084K and Q1100R) exhibited expression levels comparable to Combo 1, suggesting that these additional substitutions did not significantly further enhance expression. Combo 3, which additionally incorporates the substitutions Y246H, R413G, L653Y, T772V, and V945I, achieved a 2-fold expression increase relative to BB2.

For an in-depth manufacturability analysis of the lead candidates, with a focus on protein yield, structural integrity, and stability during purification and storage, both backbones as well as the three combinations were purified by lectin affinity chromatography followed by a polishing step using preparative SEC. Analytical SEC combined with multi-angle light scattering (SEC-MALS) analysis was performed and confirmed that the molecular weight of the proteins was consistent with a trimer (Fig 4B), where the longer retention time of Combo 3 indicated a slightly more compact spike compared to the other proteins. Next, we evaluated the melting curves of the stabilized variants using differential scanning fluorimetry (DSF). The BB1 construct exhibited a single melting event (Tm_50_) at 62.9°C (Fig 4C), similar to the Tm_50_ of BB2, which contains various substitutions aimed at enhancing expression. Notably, the Tm_50_ of the various combos showed a sequential increase: Combo 1 had a melting temperature of 69.7°C, reflecting a 6.8°C improvement compared to parental construct BB2. Additional substitutions in Combo 2 raised the melting temperature to 71.4°C, while Combo 3 exhibited the highest stability, with a melting temperature of 73.5°C. These results highlight the positive impact of the introduced substitutions on the thermal stability of S, thereby validating our initial analytical SEC screening in cell supernatant.

In addition to thermal stability, storage stability and cold chain dependence are critical attributes for vaccine candidates. Although the thermal stability experiments indicate monomer formation when spikes are incubated at temperatures near the Tm_50_ (S2 Fig), this does not accurately reflect the storage conditions for a vaccine candidate. We therefore assessed the long-term stability of the purified OC43 S designs at both 4°C and 37°C over an 8-week period. The analytical SEC profiles of the two backbone designs BB1 and BB2 as well as all three combos (Fig 4D) remained unchanged throughout this time course at both 4^o^C and 37^o^C, confirming the stability of all purified proteins.

### Structural insights into identified spike stabilizing mutations

To validate the prefusion conformation of the stabilized OC43 S designs and elucidate the mechanism underlying improved stability, we resolved the structure of OC43 Combo 3 using single particle cryo-EM. We obtained a density map at 3.1Å resolution which enabled model building for residues 15–1229 (S4 and S5 Figs). We observed additional non-protein density in the hydrophobic pocket of the S1^B^ domain, modeled as sapienic acid (S6A Fig), which was previously determined as the free fatty acid (FFA) ligand for OC43 S [20], as opposed to linoleic acid found in the analogous pocket in SARS-CoV-2 S [22].

**Fig 5:**
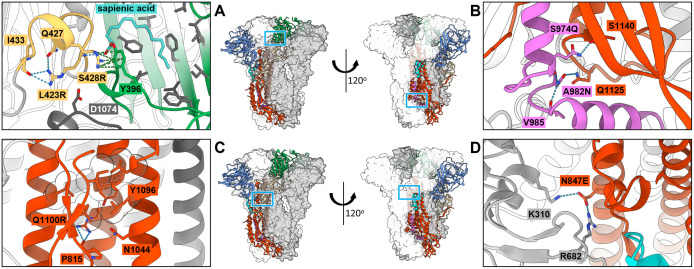
Structure of stabilized OC43 S. Model of the stabilized OC43 S Combo 3 with one protomer in cartoon representation and colored according to the schematic in Fig 1C, and two protomers (light or dark grey) in surface view (center). Regions with stabilizing substitutions are shown in close-up view: (**A**) region with L423R and S428R substitutions near the free fatty acid-binding pocket with sapienic acid bound. The loop comprising residues 422-433 is colored light orange. **(B)** Zoom in of the S974Q and A982N substitutions. **(C)** Zoomed in view of the Q1100R substitution. **(D)** Close-up view of the N847E substitution. Partial regions of the model have been omitted for clarity. Important residues are shown as sticks, oxygen atoms colored red, nitrogen atoms colored blue, hydrogen bonds shown as blue dashes and cation/aromatic ring interactions as green dashes.

The highly stabilizing L423R and S428R substitutions are situated at the inter-protomeric interface of two neighboring S1^B^ domains, near the hinge loop of the S2 central helix (Fig 5A). L423R is a space filling substitution which introduces hydrogen-bonds with the I433 backbone carbonyl and Q427 sidechain and stabilizes the 422–433 loop, including the short helix that contains the S428R substitution, while the backbone of R423 interacts via hydrogen bonding with the sapienic acid ligand. S428R, the second substitution in this region, forms an inter-protomeric cation-pi interaction with Y396, which by itself also interacts with the carboxyl group of the sapienic acid ligand via its hydroxyl group. Since FFA binding in this pocket is described to stabilize the 3 RBD down-state of the spike [20–22], we hypothesize that R428 may orient the side chain of Y396 in an optimal orientation for hydrogen bonding to the sapienic acid head group. Both arginine substitutions at positions 423 and 428 will increase S1^B^:S1^B^ interaction, lower the dissociation rate of the ligand and consequently increase spike stability by locking the S1^B^ domains in the down conformation.

S974Q and A982N are located in a region identified as the bona-fide fusion peptide in a postfusion structure of SARS-CoV-2 S [44] at the base of the prefusion spike protein (OC43 S residues 959–999) (Fig 5B). S974Q introduces a hydrogen bond with the backbone carbonyl of S1140. Since S1140 is situated in a beta-sheet that is conformationally stable between pre- to post-fusion structures, this interaction likely prevents reorganization of the fusion peptide to the extended alpha helix of HR1 in the postfusion spike. Likewise, A982N creates hydrogen bonds with the Q1125 sidechain and backbone oxygens of V985 and Q974, stabilizing the prefusion loop conformation of the fusion peptide.

The Q1100R substitution is located in the central helix of S2. The arginine sidechain that is introduced here makes a hydrogen bond with the backbone carbonyl of P815 (Fig 5C). This interaction improves packing of the HR1 region of S2 against the central helix, which otherwise undergoes a dramatic reorganization from pre- to post-fusion to an extended alpha helix.

N847E resides in a long alpha helix N-terminally of the S2’ cleavage site (Fig 5D). By substitution of the wildtype Asn for Glu, hydrogen bonds and attractive electrostatic interactions are established with K310 and R682 in S1 of a neighboring protomer. This likely stabilizes S by improving interprotomeric interactions and preventing the shedding of the S1 domain [25].

The Y246H substitution in the S1^A^ domain introduces a hydrogen bond with T227 that improves the interaction at the interprotomer interface with S1^B^ domain of a neighboring protomer, clamping the S1^B^ in the down-state, like the stabilizing sapienic acid does at the other side of S1^B^ (S8A Fig).

E838G removes the bulky glutamic acid sidechain, which allows R1058 to adopt an alternate conformation and form a hydrogen bond to A1061 (S8B Fig). Although this removes an interprotomeric hydrogen bond between E843 and R1058, it likely stabilizes the loop conformation of 1058–1065 in this region of HR1 and prevents refolding to the postfusion extended alpha-helix.

The V945I substitution is located in the fusion peptide proximal region (FPPR). Examination of this region suggests it serves as a space filling substitution improving hydrophobic interactions with I939, L942, Y948 and L1052 (S8C Fig). Since dynamics of the FPPR and interaction with the C-terminal domain of S1(CTD) have been described extensively to modulate prefusion stability of spike [42,49,50], it is likely that this substitution stabilizes S by improving FPPR packing, and interdomain interactions with S1. Moreover, we observe that the FPPR in our structure adopts a more compact conformation in which a loop comprising residues 934–941 packs more tightly against the CTD, shifting between 5.0 and 6.0Å compared to other prefusion structures of OC43 S (6NZK, 7SBX, 7PNM) [15,20,51] (S8E Fig).

Near the apex of the central helix in S2, the R1084K substitution introduces a hydrogen bond with the backbone carbonyl of A1080, improving intrahelix interactions (S7D Fig). Being located near the spike three-fold axis at the interface of three protomers, the R1084K substitution possibly also reduces repulsive interactions between the bulky positively charged sidechains.

As universally observed for CoV S maps [18,26,32,33,40,52–55], the poor resolution of the density map in the stem region prevented us from building the model for this part of S. Therefore, we could not interpret the substitutions that were introduced in this region in a structural context. Based on structure prediction of the spike stem sequence using AlphaFold3, we expect that both H1284Q and I1292K improve the coiled-coil structure by intrahelical electrostatic interactions with residues in *e* and *g* positions.

### Transfer of stabilizing substitutions to related embecoviruses

**Fig 6:**
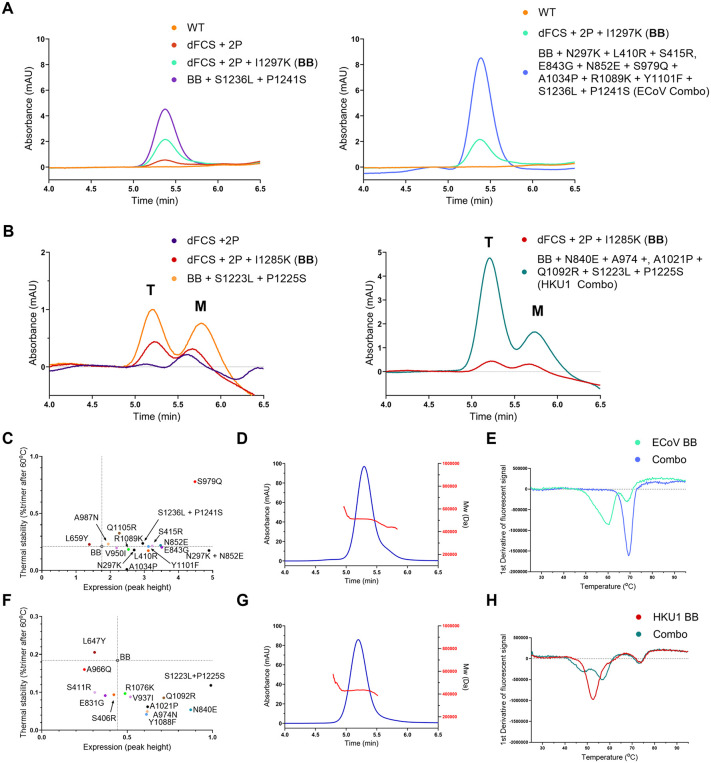
Transfer of stabilizing substitutions to related Embecoviruses. (A) Analytical SEC profiles of cell culture supernatants from Expi293F transfected with indicated ECoV S variants. **(B)** Analytical SEC of HKU1 S variants in Expi293F supernatants. Peaks corresponding to S trimers (T) and monomers (M) are indicated. **(C)** Scatter plot of expression and thermal stability of transferred substitutions in ECoV S BB. **(D)** SEC-MALS analysis of purified ECoV S Combo. **(E)** Melting temperature determination by DSF on purified protein of ECoV BB (green) and stabilized Combo (blue). **(F)** Scatter plot of expression and thermal stability of transferred substitutions in HKU1 S BB. **(G)** SEC-MALS analysis of purified HKU1 S Combo. **(H)** Melting temperature determination by DSF on purified protein of HKU1 BB (crimson) and stabilized Combo (teal).

Considering the substantial sequence homology and expected structural and functional similarities among embecovirus S fusion proteins, it is likely that the regions contributing to structural instability are conserved. We therefore presume that the stabilization approaches developed for the prefusion OC43 S protein are also relevant to other embecovirus spike proteins. We investigated whether the stabilizing substitutions of OC43 S could be transferred to the closely related ECoV S protein (78% sequence identity) and the more distantly related HKU1 S protein (63% sequence identity). First, backbone constructs with conceptually identical stabilizing elements as OC43 S BB1 were generated. ECoV and HKU1 S proteins containing furin cleavage site removal, 2P substitution (A1080P and L1081P in ECoV S, and N1067P and L1068P in HKU1 S) and C-terminal helix stabilization at the homologous I1292K position (I1297K and I1285K respectively) yielded backbones with sufficient expression for both viruses and which were consistent with the formation of trimers as based on analytical SEC analysis (Fig 6A and 6B, left panels). Strikingly, the I1285K substitution allowed detection of HKU1 S trimers, whereas a construct with removal of the furin cleavage site and 2P showed expression of an exclusively monomeric fraction with a retention time of around 5.6 minutes (Fig 6B, left panel). These constructs, designated BB, were deemed suitable to evaluate the transfer of stabilizing substitutions from OC43 S.

Given its relatively simple structural characteristics, the HR2 stem region is a prime candidate for transfer of stabilizing substitutions. Alignments of the stem regions from various embecovirus fusion proteins highlighted similar conserved imperfections as we observed in OC43 S (Fig 2A). As predicted, when the same optimization strategy was applied by introducing S1236L and P1241S in ECoV S and S1223L and P1225S in HKU1 S we observed increased trimer expression in both cases (Fig 6A, 6B respectively), confirming the approach as a general stabilization strategy.

Next, we analyzed the selected panel of tissue-culture adaptation and ReCaP substitutions from OC43 for their relevance in ECoV and HKU1 S, focusing on those located in structurally conserved regions in S. This resulted in 11 candidates selected to test for applicability in ECoV and HKU1 S (S2 Table). In addition, we included the homologous substitutions of A942P in SARS-CoV-2 [32,33] which translate to A1034P and A1021P in ECoV and HKU1 S, respectively.

Expression and thermal stability analysis of the substitutions in the ECoV BB by analytical SEC showed increases in S expression for 10 out of 11 substitutions, indicating that most substitutions can indeed be transferred to ECoV S (Fig 6C). The biggest effects were observed for S979Q, which increased thermal stability of ECoV S similarly as to what was observed for the homologous S974Q substitution in OC43 S, suggesting that the structural instability in this region is conserved between both viral S proteins. Based on the results for OC43 S (S3 Fig) we excluded non-synergistic substitutions and selected N297K, L410R, S415R, E843G, N852E, S979Q, A1034P, R1089K and Y1101F. Combining this panel with HR2 stem optimizing substitutions S1236L and P1241S in the BB background yielded the stabilized lead candidate (ECoV Combo) which showed a near 5-fold increased expression compared to BB (Fig 6A, right panel). Purification of the ECoV S Combo yielded 23.8 mg/L and determination of the molecular weight by SEC-MALS analysis confirmed the formation of trimers (Fig 6D). Furthermore, the Tm_50_ of the stabilized combination indicated a strongly improved thermal stability. While the BB melting curve revealed two distinct melting events, one major event at 59.8°C and a minor one at 68.6°C, the stabilized Combo showed a single event at 69.2°C (Fig 6E).

Similarly, we tested if the 11 substitutions were transferable to the spike of HCoV-HKU1 by determining the effect on expression and thermal stability in analytical SEC (Fig 6F). Six out of the 11 substitutions; N840E, V937I, A974N, R1076K, Y1088F, Q1092R as well as the A942P-derived A1021P substitution, showed increases in the trimer peak that was measured by analytical SEC (Fig 6F), however, none showed improved thermal stability in heat-SEC analysis. The most impactful mutations that we identified in the OC43 spike; L423R, S428R and S974Q (respectively S406R, S411R and A966Q in HKU1 S) did not translate successfully, which can be attributed to structural diversity between the OC43 and HKU1 spike in these regions.

The stabilized combination for HKU1 S (HKU1 Combo) incorporated substitutions N840E, A974N, A1021P, Q1092R, S1223L and P1225S in the HKU1 S BB and showed approximately 10-fold higher trimer expression (Fig 6B, right panel). Moreover, the trimer to monomer ratio of the stabilized combination (5:2) was improved compared to BB (3:2 trimer:monomer). Purification of HKU1 Combo yielded 20.5 mg/L S trimer as confirmed by SEC-MALS (Fig 6G). As expected from the analytical SEC screening of individual substitutions, the effect on thermal stability as determined by DSF was less pronounced compared to ECoV S; the HKU1 S BB showed a single melting event at 52.4°C, whereas for the stabilized combo we observed two melting events at 48.4°C and 56.2°C (Fig 6H). While the temperature of the major melting event was improved by 3.8°C, these results suggest that the translation of substitutions to HKU1 S do not confer the same improvements in thermal stability as were observed for ECoV and OC43 S.

### Structural characterization of prefusion-stabilized ECoV S

To confirm that the transferred mutations improve stability through similar mechanisms as in OC43 S and determine the first structure of a prefusion ECoV spike, we performed single particle cryo-EM analysis on the ECoV S stabilized Combo. The dataset resolved to a global resolution of 2.8Å, which allowed for model building of the major part of residues 15–1234 (S4 and S5 Figs). Overall, the determined structure of the ECoV spike shows similar architecture as other betacoronavirus spikes in the prefusion conformation [15,17,25,26,30,56]. The S1 subunit sits atop the S2 subunit in a typical V-shaped architecture, due to the S1^B^ domain of one protomer folding on top of the S2 subunit of a neighboring protomer. As in the OC43 S map, we observed non-protein density in the homologous hydrophobic pocket of the ECoV S1^B^ domain (S6B Fig), which we interpreted as sapienic acid —consistent with production of the spike protein in a human cell expression system.

**Fig 7:**
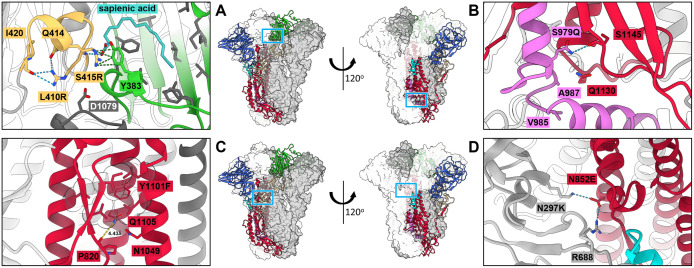
Structure of stabilized ECoV S. Model of the stabilized ECoV S Combo with one protomer in cartoon representation and colored according to the schematic in Fig 1C, and two protomers (light or dark grey) in surface view (center). Regions with stabilizing substitutions that are discussed are shown in close-up view: (**A**) region with L410R and S415R substitutions near the free fatty acid-binding pocket with sapienic acid bound. The loop comprising residues 409-420 is colored light orange. **(B)** Zoom in of the S979Q substitution. **(C)** Zoomed in view of the Y1101F substitution. F1101 and nearby hydrophobic residues are shown in stick and sphere representation. **(D)** Close-up view of the N852E and N297K substitutions. Partial regions of the model have been omitted for clarity. Important residues are shown as sticks, oxygen atoms colored red, nitrogen atoms colored blue, hydrogen bonds shown as blue dashes, atomic distances indicated with yellow dashes and cation/aromatic ring interactions as green dashes.

When we inspect the substitutions that were translated to ECoV S, we observe a similar mechanism of stabilization. The L410R and S415R substitutions in the S1^B^ domain confer stability through conserved elements; the R410 sidechain forms a hydrogen bond network with the backbone atoms of Q414 and I420, while the R410 backbone carbonyl interacts with the sapienic acid ligand. In addition, the R415 sidechain is oriented to make cation-pi interactions with Y383 to enforce hydrophilic interactions with the fatty acid head group (Fig 7A).

The determined structure also revealed that similarly to S974Q in OC43, Q979 in the ECoV S structure contacts the backbone carbonyl of S1145 (Fig 7B). Likely stabilizing the fusion peptide in the prefusion state. Although the A987N substitution was not introduced in the ECoV S Combo due to the modest increase in expression that was observed in analytical SEC, the structural conservation in this region suggests that it may stabilize ECoV S synergistically with S979Q as we determined in the OC43 spike.

While Q1105R and Y1101F were both successful in improving ECoV S expression, we opted to include only the latter in the ECoV S stabilized Combo since these substitutions were determined to be mutually exclusive in OC43 S (S3 Fig). In ECoV S, Y1101F improves stability by increasing hydrophobic interactions with nearby hydrophobic side chains (Fig 7C) which would be less favorable for the wildtype Tyr due to its hydroxyl group.

Due to the structural conservation at residue 847 in the OC43 spike, we are able to apply the understanding gained from N847E to the homologous region in the ECoV spike. Alignment of the ECoV S amino acid sequence and predicted structure with OC43 S showed that while R682 in OC43 is conserved, K310 is replaced by N at the homologous 297 position in ECoV (S10A Fig). By introducing substitutions N852E and N297K, we reconstitute the stabilizing interactions with R688 and K297 (Fig 7D). Further supporting our reasoning, analytical SEC showed that each individual mutation improved expression of ECoV S in supernatant of transfected Expi293 cells, but the double substitution N297K + N852E performed best (S10B Fig).

## Discussion

The continuous emergence of zoonotic coronaviruses underscores the need for cross-species applicable strategies to stabilize spike proteins for vaccine design. By integrating rational design, phylogenetic analysis, and machine-learning, we developed a versatile framework for engineering prefusion-stabilized embecovirus spike proteins. The substitutions suggested by ReCaP highlights the model’s ability to improve the geometry of complex polar and charged interactions. Although not all the identified substitutions translated uniformly as a one-size-fits-all approach, collectively, the panel of stabilizing substitutions that we identified in OC43 S provides a generalizable design framework that allows stabilization of S antigens of related embecoviruses, as demonstrated for ECoV and HKU1 S proteins.

One of the strategies was the stabilization of the HR2 coiled-coil regions. By replacing suboptimal residues in the heptad repeats, we significantly improved expression across multiple embecovirus spikes. The transferability of these modifications was further exemplified by the increased expression and stability of alpha- and delta-CoV spikes carrying similar heptad optimizing substitutions (S1 Fig). AlphaFold3 models support that the substitutions in this region improve coiled-coil packing, likely promoting trimer formation. Similar strategies have improved prefusion stability of the fusion proteins of HMPV [48], Nipah, respirovirus types 1 and 3 [57], and RSV [37] by targeting the HR2 stem. By improving coiled-coil interactions at the stem trimer interface our approach enables the design of trimeric coronavirus prefusion spikes without relying on heterologous trimerization domains (e.g., GCN4 or foldon), thereby reducing the risk of inducing non-specific immune responses [37].

Many of our most potent stabilizing substitutions were identified through ReCaP. In line with previous findings [48], a large number of successful hydrophilic substitutions were identified using this approach, further exemplifying the usefulness of microenvironment-based predictions for protein stability enhancement. Key substitutions were located in the fusion peptide (S974Q), preventing reorganization to the postfusion extended alpha-helical bundle, and at the S1^B^-S1^B^ interface (L423R and S428R), where substitutions improve interprotomeric contacts mediated by the FFA ligand to lock the 3-down conformation. Interestingly, ReCaP was able to stabilize this region despite the absence of the FFA in the structural model used (PDB ID: 6NZK). Our findings suggest that release of the S1^B^ domain, release of the fusion peptide, and dissociation of the HR2 coiled-coil are key determinants of prefusion spike metastability. Studies focused on designing prefusion-stabilized SARS-CoV-2 spike proteins have reported stabilizing substitutions in regions analogous to those found here for embecoviruses. For example, A899Q [58], A892P and A899P [32,33] in the fusion peptide, were found to have a stabilizing effect on the SARS-CoV-2 spike, similar to the S974Q and S979Q substitutions described here for OC43 and ECoV S. Additional strategies using disulfide-bonds [59], or chemical-crosslinking [60] have likewise improved prefusion stability by locking the S1^B^ domains in the all-down conformation.

The substitutions near the FFA binding pocket in OC43 and ECoV improved stability by interacting with the conserved FFA binding motif [23]. However, HKU1 S has lost binding of FFA in this pocket, which likely explains why S406R and S411R proved ineffective in increasing prefusion stability in HKU1 S. Further stabilization of HKU1 S, will therefore require a dedicated ReCaP analysis based on its specific structure, while the mechanistic insight gained from the successful ReCaP substitutions may guide further rational design in the most critical regions of instability of HKU1 and other coronavirus spikes in general.

In this study we present the first structure of the Equine coronavirus spike protein. The stabilized OC43 and ECoV spikes show a high degree of structural similarity, with an overall root mean square deviation value of 1.26 Å. The greatest structural variation is observed in S1^B^ (67% sequence identity) specifically at the tip of the domain (S9 Fig). Since these loops are commonly involved in receptor-binding in spike proteins of other coronaviruses [61–66], this may hint at unique specificity for a putative proteinaceous receptor for both OC43 and ECoV.

In conclusion, this work presents a cross-species applicable strategy for prefusion stabilization of coronavirus spike proteins. The insights gained here not only improve the design of embecovirus immunogens but also offer general principles for engineering metastable viral glycoproteins, which are critical for future pandemic preparedness.

## Materials and methods

### Protein expression and purification

Codon-optimized constructs encoding spike ectodomains for OC43 (residues 1–1297, GenBank: ANZ78834.1), ECoV (1–1302, consensus sequence [36] of GenBank accessions: AAQ67205.1, BAJ52885.1, UVD39588.1 and BAS18866.1), HKU1 (1–1290, GenBank: Q0ZME7.1), NL63 (1–1291, GenBank: ARB07399.1), 229E (1–1108, GenBank: KY983587.1), and Hu-PDCoV (1–1092, GenBank: MW685622.1) were synthesized, inserted into the pCDNA3.1 vector and maxiprepped by Genscript (Piscataway, NJ, USA). Phylogenetic analysis (see S1 Table), the machine-learning algorithm ReCaP (described previously [48]) and rational design were used to select amino acid substitutions which were cloned into the above plasmids by Genscript (Piscataway, NJ, USA). For initial expression screening, Expi293 cells in 96-well plates (200 µL per well) were transfected with the ExpiFectamine 293 Transfection Kit (Gibco, Thermo Fisher Scientific) following the supplier’s instructions. Cells were cultured in Expi293F Expression medium [+] GlutaMAX (Gibco, Thermo Fisher Scientific) with 100 U/mL Pen-Strep (Gibco) under controlled conditions (37°C, 8.0% CO₂, 75% humidity, 250 rpm) for three days, after which supernatants were collected by centrifugation and 0.2 µm filtration (Pall Life Sciences). These clarified supernatants were stored at 4°C before analysis using various assays.

For protein purification purposes, Expi293F cells were transfected in a similar manner, but at 300 mL scale and cultured for five days. Supernatants were harvested, clarified by centrifugation, and filtered through a 0.22 µm PES filter (Nalgene). Following harvest, S proteins were purified from culture supernatants through a two-step *Galantus nivalis* lectin-based protocol on an ÄKTA Avant 25 system (GE Healthcare). Clarified supernatant was applied to a Tricorn 10/50 column (Cytiva) packed with 4 mL of *Galanthus nivalis* lectin agarose (Vector Laboratories) pre-equilibrated with 40 mM Tris, 500 mM NaCl, pH 7.4. Elution of bound proteins was achieved using equilibration buffer supplemented with 1 M methyl alpha-D-mannopyranoside. The eluted fractions were subsequently concentrated using an Amicon Ultra 15 filter with a 100-kDa molecular weight cutoff (Millipore). For final purification, the protein was polished using Size Exclusion Chromatography (SEC) on a HiLoad Superdex 200 16/600 column (GE Healthcare) equilibrated in 20 mM Tris, 150 mM NaCl, pH 7.4. Peak fractions were collected, pooled, and filtered through a Millex-GV 0.22 µm filter membrane (Millipore Sigma). The purified protein products were either stored at 4°C or snap frozen in liquid N2 and stored at -80°C before analysis using various assays.

### Analytical size exclusion chromatography

Analytical Size Exclusion Chromatography (SEC) and Multi-Angle Light Scattering (MALS) were conducted on an ultra-high-performance liquid chromatography (UHPLC) Vanquish system (Thermo Scientific) equipped with a µDAWN TREOS MALS instrument, an Optilab µT-rEX Refractive Index (RI) Detector, and an in-line Nanostar DLS reader (Wyatt Technology). Cleared supernatants were loaded onto a SRT-C SEC-500 15 cm column with the corresponding guard column (Sepax Technologies) and equilibrated in running buffer (150 mM sodium phosphate, 50 mM NaCl, pH 7.0) with a flow rate of 0.35 mL/min. For supernatant samples, µMALS detectors were set offline, and SEC data were analyzed with Chromeleon software (Thermo Fisher Scientific). Baseline corrected UV signals from clarified supernatants of cells transfected with an empty pcDNA3.1 plasmid were subtracted from those of spike-transfected samples to control for background protein levels. For purified protein analysis, µMALS detectors were in-line, allowing comprehensive SEC-MALS analysis via Astra software (Wyatt Technology), where dn/dc values of 0.1850 for protein and 0.1410 for glycans were applied. The RI detector provided concentration data for molecular weight calculations, while UV signals were used for assessing mass recovery.

### Thermal stability using analytical SEC (Heat-SEC)

To assess thermal stability, clarified cell culture supernatants were subjected to heat treatments at various temperatures (60°C or 64°C) for 15 minutes, while control samples were kept at 4°C. For each temperature, 50 μL of supernatant was aliquoted in a 96-well PCR plate (Eppendorf), sealed and incubated in an Eppendorf ThermoMixer C. Following incubation, samples were centrifuged at 10,000 x g for 10 minutes to remove precipitates. Analytical SEC was then performed by injecting 20 μL of each sample onto a SRT-C SEC-500 15 cm column (Sepax Technologies) with corresponding guard column, equilibrated in 150 mM sodium phosphate, 50 mM NaCl, pH 7.0, and run at 0.35 mL/min at 25°C on a Vanquish ultra-high-performance liquid chromatography system (Thermo Scientific). Data analysis was conducted using the Chromeleon 7.2.8.0 software package.

### Differential scanning fluorimetry

To assess protein stability, differential scanning fluorimetry (DSF) was performed using 20 µg of purified protein diluted in 90 µL of PBS (pH 7.4, Gibco) and mixed with 10 µL of a 50x working solution of SYPRO Orange fluorescent dye (5000x stock, Invitrogen). Triplicate 30 µL aliquots of this mixture were loaded into a MicroAmp Fast Optical 96-well plate (ThermoFisher Scientific), sealed with MicroAmp Optical Adhesive Film (ThermoFisher), and a dye-only sample was included as a negative control for reference subtraction. Measurements were carried out on an Applied Biosystems ViiA 7 qPCR instrument, where temperature was gradually increased from 25°C to 95°C at 0.015°C per second. Data acquisition was continuous, recording the reporter signal (ROX), with melting temperatures (T_m_) derived from the minimum point of the negative first derivative plot of fluorescence as a function of temperature.

### Cryo-EM sample preparation and data collection

Immediately before blotting and plunge freezing, 1 µL of 0.2% (w/v) fluorinated octyl maltoside (FOM) was added to 9 µL of purified protein sample of OC43 (1.1 mg/mL) or ECoV (1.2 mg/mL) spike trimer resulting in a final FOM concentration of 0.02% FOM (w/v). 3.5 µL of the protein/detergent mixture was then applied to glow-discharged (15 mA, 20 sec, Pelco easiGlow) Quantifoil R1.2/1.3 Cu 200 mesh grids (Quantifoil Micro Tools GmbH), blotted for 3.5 sec using 0° blot force at 100% humidity and plunge frozen into liquid ethane using Vitrobot Mark IV (Thermo Fisher Scientific). The data were collected on a Thermo Fisher Scientific Krios G4 Cryo Transmission Electron Microscope (Cryo-TEM) equipped with a K3 Direct Electron Detector (Thermo Fisher Scientific) and Bioquantum energy filter (Gatan) operated at 300 kV at the Netherlands Centre for Electron Nanoscopy (NeCEN). In total 4,020 and 2,357 movies were collected for ECoV and OC43 S, respectively, at a nominal magnification of ×105,000, corresponding to a calibrated pixel size of 0.836 Å/pix over a defocus range of −1.0 to −2.5 μm. To mitigate observed orientation bias of ECoV and OC43, these data sets were collected under a stage tilt of 33°. A full list of data collection parameters can be found in S3 Table.

### Cryo-EM single particle image processing

Data processing was performed using the CryoSPARC Software package [67]. After patch-motion and CTF correction, particles were picked using a blob picker, extracted at 6x binning and subjected to 2D classification. Following initial 2D classification, particles belonging to class averages that displayed high-resolution detail were selected for ab-initio reconstruction into four classes.

Particles belonging to the representative trimeric spike complex class were re-extracted at 1.4x binning. Spikes were subjected to non-uniform refinement with optimization of per-group CTF parameters [68]. At this point, the global resolution of the spikes was 3.2 Å for ECoV and 3.6 Å for OC43. The resulting volumes were then used to perform template picking on the data sets. The picked particles were extracted at 4x (ECoV) or 5x (OC43) binning and subjected to 2D classification. Selected 2D class particles were then re-extracted at 1.6x (ECoV) or 1.3x (OC43) binning and selected for ab-initio reconstruction into four classes. Particles belonging to the representative trimeric spike classes were then subjected to non-uniform refinement. Finally, reference-based motion correction was performed on the final particle stacks of both data sets, improving the resolution to a final resolution of 2.8 Å for ECoV S (with optimization of per-group CTF parameters) and 3.1 Å for OC43 S (with optimization of per-particle defocus).

### Model building and refinement

UCSF ChimeraX [69] (version 1.9) and Coot [70] (version 0.9.8) were used for model building. The structure of the previously reported OC43 spike glycoprotein (PDB ID 6NZK) [15] and AlphaFold3 [45] generated S1^B^ domain (residues 341–614) of the OC43 spike was used as a starting point, which was then mutated to the correct amino acid sequence. Models were individually rigid body fitted into the density map using the UCSF ChimeraX “Fit in map” tool and then combined. Initial model building in the ECoV spike density map was conducted using ModelAngelo [71]. ELBOW [72] was used to generate ligand restraints for the sapienic acid molecule. The resulting models were then edited in Coot using the ‘real-space refinement, carbohydrate module [73] and ‘sphere refinement’ tools. Additional manual fitting was done using the ISOLDE [74] package in ChimeraX to address rotamer, bond angle and Ramachandran outliers. Following this, iterative rounds of manual fitting in Coot and real space refinement in Phenix [75] were carried out to improve rotamer, bond angle and Ramachandran outliers. During refinement with Phenix, secondary structure and non-crystallographic symmetry restraints were imposed. The final model was validated with MolProbity [76,77], and fitted glycans validated using Privateer [78].

For a more detailed processing methodology, see S4 and S5 Figs.

## Supporting information

S1 Fig HR2 stem optimization stabilizes spike proteins of coronaviruses from the alpha- and delta-coronavirus genus. Analytical SEC profiles of backbones of NL63, 229E and Hu-PDCoV S proteins (top row) or with indicated substitutions (bottom row). Supernatants were measured immediately after harvesting (solid line), and after 11 days (dashed line) or 30 days (dotted line) storage at 4^o^C to determine stability of prefusion S.
(TIF)

S2 Fig Analytical SEC profiles of candidates selected from OC43 S screen. Analytical SEC data supporting analysis shown in Fig 3; supernatant of Expi293F cells transfected with OC43 S BB1 (backbone) or with the indicated substitution was harvested and heated for 15 min at 60^o^C (dashed line), 64^o^C (dotted line) or untreated (4^o^C; solid line) before analysis by analytical SEC.
(TIF)

S3 Fig Stabilizing substitutions Y1096F and Q1100R are mutually exclusive. (**A**) Analytical SEC profiles of BB2, and BB2 including single or double substitutions of Y1096F and Q1100R to determine negative or additive effects. (**B**) Scatter plot of expression (as area under the curve; AUC) and thermal stability calculated from SEC profiles in (A).
(TIF)

S4 Fig Single-particle cryo-EM data processing pipeline for the OC43 Combo 3 and ECoV Combo spike. (TIF)

S5 Fig Single-particle cryo-EM data processing for the OC43 Combo 3 and ECoV Combo spikes. (**A**) Representative motion-corrected micrograph out of ~2,357 similar micrographs of the OC43 S Combo 3 dataset. Scale bar = 50 nm. (**B**) As shown in A for the ECoV S Combo dataset containing ~4,020 similar micrographs. Scale bar = 50 nm. (**C**) Representative 2D classes for OC43 S Combo 3. (**D**) Representative 2D classes for ECoV S Combo. (E) Local resolution filtered EM density map for the refined OC43 S Combo 3, colored according to local resolution which was calculated in CryoSPARC. (F) As shown in E for the ECoV S Combo. (**G**) Angular distribution plot of the final for OC43 S Combo 3 C3 refined EM density maps. (**H**) Angular distribution plot of the final for ECoV S Combo C3 refined EM density maps. (**I**) Gold-standard Fourier shell correlation (FSC) curve generated from the independent half maps contributing to the 3.1 Å global resolution density map of the OC43 S Combo 3. (**J**) As shown in I for the 2.8 Å global resolution density map of the ECoV S Combo.
(TIF)

S6 Fig Atomic models of OC43 Combo 3 and ECoV Combo spikes fitting in EM density. **(A**) Example density of the OC43 S Combo 3 map at the interacting region of sapienic acid with Y396 and R428. EM density is shown as blue mesh (**B**) Example density of the ECoV S Combo map as in A. (**C**) Example density of the OC43 S Combo 3 map at the interacting region of the S974Q substitution. EM density shown as blue mesh, hydrogen bond between S974Q sidechain and S1140 backbone carbonyl in blue dashes. (**D**) Example density of the ECoV S Combo map as in C. Hydrogen bond between S979Q sidechain and S1145 backbone carbonyl in blue dashes.
(TIF)

S7 Fig EM density for the substitutions in OC43 and ECoV S. Map density is shown for the substituted amino acids and important interacting residues in the OC43 Combo 3 spike (left panels) and ECoV Combo spike (right panels).
(TIF)

S8 Fig Structural analysis of stabilizing substitutions in OC43 S. **(A**) Zoom in of region near V945I substitution in the fusion peptide-proximal region of the S2 domain. (**B**) E838G, (**C**) Y246H, (**D**) R1084K, view is along the three-fold axis of the spike protein. (**E**) Zoom in of how the fusion peptide-proximal region (dark blue) packs against the S1B domain of the adjacent protomer (light grey). (**F**) comparison of the fusion peptide proximal region of published OC43 S structures (6NZK, 7SBX and 7PNM) with the OC43 S Combo 3 structure. Distances between Combo 3 (dark blue) and 7PNM (orange); 6.02Å, and 6NZK (light blue); 5.04Å are indicated.
(TIF)

S9 Fig Structural similarity of models for OC43 and ECoV S. **(A**) Structural alignment of ECoV S Combo (blue) and OC43 S Combo 3 (grey) (78% sequence similarity). A single monomer is shown. (**B**) Zoom in of the tip of the S1^B^ domain.
(TIF)

S10 Fig Translation of N847E substitution to ECoV S. (**A**) Sequence alignment of OC43, HKU1 and ECoV spike proteins. Positions of K310 (blue), R682 (magenta) and N847 (green) based on OC43 S numbering are highlighted. (**B**) Supernatant analytical SEC profiles of ECoV S proteins containing single or double substitutions compared to the backbone (BB) as indicated. N852E is the homologous substitution of N847E in OC43. N297K recapitulates the K310 residue in OC43 which is important for the stabilizing interaction.
(TIF)

S1 Table Sequences of tissue-culture adapted strains of HCoV-OC43 used for phylogenetic analysis. Accession codes of OC43 spike sequences of ATCC VR-759 and other related strains which were retrieved from GenBank. The “”RRSRG” or “IRSRG” sequence at the S1/S2 furin cleavage site was used to select strains that were passaged in vitro.
(TIF)

S2 Table Translation of homologous substitutions from OC43 to ECoV and HKU1 S. Overview of the amino acid positions (Res #) at which stabilizing substitutions were introduced in OC43 (top row) and the equivalent position in the sequence of ECoV and HKU1 spikes in the same column. The wildtype amino acid at this position (WT), and the amino acid that is present in the final lead candidate (Combo) are indicated, as well as the design strategy from which the substitutions originate (“Phyl.”: Phylogenetic analysis, “AI”: ReCAP prediction, “BB”: 2P and A942P (from SARS-CoV-2) related substitutions, “HR2”: Heptad repeat 2 stabilization).
(TIF)

S3 Table Cryo-EM data collection, refinement and validation statistics. (TIF)
