# Correction: AI-guided prefusion stabilization of the human coronavirus OC43 spike protein enables universal embecovirus antigen design

**DOI:** 10.1371/journal.ppat.1014550

**Published:** 2026-08-27

**Authors:** The PLOS Pathogens Staff

There are errors in the Supporting Information Figures and Tables. Please view the correct S1–S10 Figs and S1–S2 Tables below.

The publisher apologizes for the errors.

## Supporting information

S1 Fig HR2 stem optimization stabilizes spike proteins of coronaviruses from the alpha- and delta-coronavirus genus. Analytical SEC profiles of backbones of NL63, 229E and Hu-PDCoV S proteins (top row) or with indicated substitutions (bottom row). Supernatants were measured immediately after harvesting (solid line), and after 11 days (dashed line) or 30 days (dotted line) storage at 4^o^C to determine stability of prefusion S.
(TIF)

S2 Fig Analytical SEC profiles of candidates selected from OC43 S screen. Analytical SEC data supporting analysis shown in Fig 3; supernatant of Expi293F cells transfected with OC43 S BB1 (backbone) or with the indicated substitution was harvested and heated for 15 min at 60^o^C (dashed line), 64^o^C (dotted line) or untreated (4^o^C; solid line) before analysis by analytical SEC.
(TIF)

S3 Fig Stabilizing substitutions Y1096F and Q1100R are mutually exclusive. (**A**) Analytical SEC profiles of BB2, and BB2 including single or double substitutions of Y1096F and Q1100R to determine negative or additive effects. (**B**) Scatter plot of expression (as area under the curve; AUC) and thermal stability calculated from SEC profiles in (A).
(TIF)

S4 Fig Single-particle cryo-EM data processing pipeline for the OC43 Combo 3 and ECoV Combo spike. (TIF)

S5 Fig Single-particle cryo-EM data processing for the OC43 Combo 3 and ECoV Combo spikes. (**A**) Representative motion-corrected micrograph out of ~2,357 similar micrographs of the OC43 S Combo 3 dataset. Scale bar  =  50 nm. (**B**) As shown in A for the ECoV S Combo dataset containing ~4,020 similar micrographs. Scale bar  =  50 nm. (**C**) Representative 2D classes for OC43 S Combo 3. (**D**) Representative 2D classes for ECoV S Combo. (E) Local resolution filtered EM density map for the refined OC43 S Combo 3, colored according to local resolution which was calculated in CryoSPARC. (F) As shown in E for the ECoV S Combo. (**G**) Angular distribution plot of the final for OC43 S Combo 3 C3 refined EM density maps. (**H**) Angular distribution plot of the final for ECoV S Combo C3 refined EM density maps. (**I**) Gold-standard Fourier shell correlation (FSC) curve generated from the independent half maps contributing to the 3.1 Å global resolution density map of the OC43 S Combo 3. (**J**) As shown in I for the 2.8 Å global resolution density map of the ECoV S Combo.
(TIF)

S6 Fig Atomic models of OC43 Combo 3 and ECoV Combo spikes fitting in EM density. **(A**) Example density of the OC43 S Combo 3 map at the interacting region of sapienic acid with Y396 and R428. EM density is shown as blue mesh (**B**) Example density of the ECoV S Combo map as in A. (**C**) Example density of the OC43 S Combo 3 map at the interacting region of the S974Q substitution. EM density shown as blue mesh, hydrogen bond between S974Q sidechain and S1140 backbone carbonyl in blue dashes. (**D**) Example density of the ECoV S Combo map as in C. Hydrogen bond between S979Q sidechain and S1145 backbone carbonyl in blue dashes.
(TIF)

S7 Fig EM density for the substitutions in OC43 and ECoV S. Map density is shown for the substituted amino acids and important interacting residues in the OC43 Combo 3 spike (left panels) and ECoV Combo spike (right panels).
(TIF)

S8 Fig Structural analysis of stabilizing substitutions in OC43 S. **(A**) Zoom in of region near V945I substitution in the fusion peptide-proximal region of the S2 domain. (**B**) E838G, (**C**) Y246H, (**D**) R1084K, view is along the three-fold axis of the spike protein. (**E**) Zoom in of how the fusion peptide-proximal region (dark blue) packs against the S1B domain of the adjacent protomer (light grey). (**F**) comparison of the fusion peptide proximal region of published OC43 S structures (6NZK, 7SBX and 7PNM) with the OC43 S Combo 3 structure. Distances between Combo 3 (dark blue) and 7PNM (orange); 6.02Å, and 6NZK (light blue); 5.04Å are indicated.
(TIF)

S9 Fig Structural similarity of models for OC43 and ECoV S. **(A**) Structural alignment of ECoV S Combo (blue) and OC43 S Combo 3 (grey) (78% sequence similarity). A single monomer is shown. (**B**) Zoom in of the tip of the S1^B^ domain.
(TIF)

S10 Fig Translation of N847E substitution to ECoV S. (**A**) Sequence alignment of OC43, HKU1 and ECoV spike proteins. Positions of K310 (blue), R682 (magenta) and N847 (green) based on OC43 S numbering are highlighted. (**B**) Supernatant analytical SEC profiles of ECoV S proteins containing single or double substitutions compared to the backbone (BB) as indicated. N852E is the homologous substitution of N847E in OC43. N297K recapitulates the K310 residue in OC43 which is important for the stabilizing interaction.
(TIF)

S1 Table Sequences of tissue-culture adapted strains of HCoV-OC43 used for phylogenetic analysis. Accession codes of OC43 spike sequences of ATCC VR-759 and other related strains which were retrieved from GenBank. The “”RRSRG” or “IRSRG” sequence at the S1/S2 furin cleavage site was used to select strains that were passaged in vitro.
(PPTX)

S2 Table Translation of homologous substitutions from OC43 to ECoV and HKU1 S. Overview of the amino acid positions (Res #) at which stabilizing substitutions were introduced in OC43 (top row) and the equivalent position in the sequence of ECoV and HKU1 spikes in the same column. The wildtype amino acid at this position (WT), and the amino acid that is present in the final lead candidate (Combo) are indicated, as well as the design strategy from which the substitutions originate (“Phyl.”: Phylogenetic analysis, “AI”: ReCAP prediction, “BB”: 2P and A942P (from SARS-CoV-2) related substitutions, “HR2”: Heptad repeat 2 stabilization).
(PPTX)

## References

[1] Melchers JM, Juraszek J, Hulswit RJG, van Overveld D, Le L, van Kuppeveld FJM, et al. AI-guided prefusion stabilization of the human coronavirus OC43 spike protein enables universal embecovirus antigen design. PLoS Pathog. 2026;22(3):e1013998. doi: 10.1371/journal.ppat.1013998 41871159 PMC13035233

